# Genetic Epidemiology of Tuberculosis Susceptibility: Impact of Study Design

**DOI:** 10.1371/journal.ppat.1001189

**Published:** 2011-01-20

**Authors:** Catherine M. Stein

**Affiliations:** Department of Epidemiology and Biostatistics, and Tuberculosis Research Unit, Case Western Reserve University, Cleveland, Ohio, United States of America; University of California San Diego, United States of America

## Abstract

Several candidate gene studies have provided evidence for a role of host genetics in susceptibility to tuberculosis (TB). However, the results of these studies have been very inconsistent, even within a study population. Here, we review the design of these studies from a genetic epidemiological perspective, illustrating important differences in phenotype definition in both cases and controls, consideration of latent *M. tuberculosis* infection versus active TB disease, population genetic factors such as population substructure and linkage disequilibrium, polymorphism selection, and potential global differences in *M. tuberculosis* strain. These considerable differences between studies should be accounted for when examining the current literature. Recommendations are made for future studies to further clarify the host genetics of TB.

## Introduction

Tuberculosis (TB), caused by *Mycobacterium tuberculosis* (Mtb), is a growing public health problem in the era of the HIV/AIDS pandemic. Among the one-third of the world infected by Mtb [1], almost 8 million new cases of TB occur annually, with 2 million deaths attributed to the disease each year. Only 10% of those individuals infected by Mtb go on to develop clinical disease, and disease presentation itself is heterogeneous, suggesting that host factors play a large role in disease susceptibility and natural history. An increased understanding of the host response to Mtb will facilitate the development of new vaccines and therapeutics [2].

Several studies have suggested a role for host genetics in TB susceptibility. Support for genetic susceptibility to TB in humans was first provided by twin studies [3], [4], animal models [5]–[8], then later segregation analyses [9], [10]. Countless candidate gene studies have been conducted, as well as seven genome-wide linkage scans [11]–[17]. However, there is a great deal of inconsistency across these studies. Among studies of any candidate gene, there are always several reports that provide both positive and negative evidence for an association with TB. Within genome scans, there has been replication of some results across two of the studies [14], [15], but there is very little replication across the remaining papers.

There are a number of key components of the design of these studies that may explain the inconsistency in the literature. The objective of this review is to discuss these issues, illustrated with examples from the TB genetics literature, and propose some approaches for taking a more thorough approach to the study of TB genetics.

## Impact of Study Design

### Phenotype Definition

The first step in any epidemiological study is to define the criteria used to diagnose disease. Then, one must define what is meant by non-diseased individuals (“controls”). In TB, this is complicated, because the pathogenesis of TB can be thought of as a two-stage process [18]. The first stage consists of latent Mtb infection (LTBI), in which Mtb establishes a productive infection but does not produce symptoms. LTBI is diagnosed by a positive tuberculin skin test (TST) and/or positive interferon-γ response assay (IGRA) in the absence of clinical signs and symptoms of full-blown disease [19], [20]. Definitive diagnosis of pulmonary TB requires the recovery of Mtb from sputum and cultivation in culture or detection of acid-fast bacilli (AFB) on smear [19], [21]. Studies have shown that AFB smear is less sensitive than culture, and that AFB smear grade could reflect differences in disease severity [21]. Smear-negative, culture-positive TB is also a problem in developing countries [21]. Thus, the method used to diagnose TB could affect the comparability of studies, and these differences could reflect variation in disease severity or even potential misclassification of disease status, generating a significant impact on the type I and type II error of studies. Here, we will first review the various diagnostic criteria used for TB disease, then the clinical characterization of study controls, and how these differences in study design may affect the interpretation of results across studies.

As stated by Möller and colleagues [22], studies of TB are “exquisitely sensitive to phenotype definition”. Different criteria have been used to diagnose TB in different study sites. Here, we focus on studies of the *NRAMP1* (*SLC11A1*) gene, which has been studied most extensively (Table 1). To summarize, some studies have used the gold standard definition for TB diagnosis based on growth of Mtb in culture [19], though other studies only diagnosed TB patients based on positive AFB smear. Some studies had heterogeneous diagnostic criteria, classifying together cases diagnosed by smear or culture or symptoms. Other studies have combined pulmonary and extrapulmonary TB cases in the analysis [23]–[27]. Notice that of the 12 studies demonstrating an association between *NRAMP1* and TB, only four used culture positivity as their diagnosis method. Could these differences in diagnostic criteria disguise differences in disease severity across populations?

**Table 1:** Summary of TB association genetic studies of NRAMP1/SLC11A1, including TB diagnostic criteria, characterization of controls, and whether there was an association with any SNP in the gene.

Population (Reference)	TB Diagnostic Criteria	Characterization of Controls	Association?
Gambia [80]	Smear +	Healthy blood donors	Yes
Gambia [81]	Smear +	Healthy blood donors	
Malawi [82]	Smear + OR culture + OR histology	Unrelated with no history of infectious disease	Yes
Morocco [37]	Culture +	Healthy family members	
Tanzania [83]	Culture +	Blood donors	Yes
Guinea [36]	Microscopy (smear +? Culture +?)	Unaffected relatives	
South Africa [32]	Smear + OR culture +	Unrelated healthy	Yes
Caucasian and African American [26]	Culture + OR past diagnosis	Household members in close contact	Yes
Caucasian [84]	Culture + OR response to TB treatment	Clinic patients without infectious disease	Yes
Caucasian, African American, and Asian [27]	Culture +	Tuberculin skin test positive	
Cambodia [85]	Smear +	Hospital/clinic patients	Yes
China [86]	Smear + OR culture + OR symptoms and radiological evidence; males only	Unrelated healthy males	Yes
Japan [87]	Smear + OR culture +	No history of TB disease	Yes
Japan [88]	Smear +	Random clinic patients	Yes
Taiwanese [89]	Culture +	Clinic patients without pulmonary disease	
Japan [90]	Smear +	Healthy blood donors without history of pulmonary or inflammatory disease	
Thai [91]	Culture +	Healthy blood bank donors	
China [92]	Culture +	Hospital patients and healthy blood donors	Yes
Korea [93]	Culture + (unclear)	No history of TB disease	Yes
Japan [94]	Smear + OR culture +	Unrelated healthy	
Poland [38]	Culture +	TST negative	

Smear + refers to AFB smear positive. “Culture +” could include more stringent definitions such as culture positive, smear positive, and radiological evidence consistent with TB.

This table is limited to studies published in English so that case and control definitions could be determined. It is also limited to studies of pulmonary TB in all age groups.

Related to this is the definition of controls. It is unknown in many of these studies whether or not the “controls” were latently infected with Mtb, as evidenced by either a TST or IGRA. Recent studies have suggested some genes may actually be related to LTBI and not progression to TB [15], [28], [29], while other studies have suggested some genes may differentiate between LTBI and active TB disease [30], [31]. This is important in truly understanding the role of these genes in disease pathogenesis and progression. If controls are latently infected, and there is an association seen between a gene and TB, that suggests the gene influences progression from LTBI to TB. However, if controls are uninfected, it is unclear whether an association implies susceptibility for developing active disease or just acquisition of LTBI.

Finally, the selection of controls is not trivial. In a case-control study, controls should be similar to cases in every way possible except for the presence of disease. In studies of TB, this means controls should be exposed to infectious TB cases, so that they have the opportunity to acquire infection and then progress to active TB disease. Some studies conducted in TB-endemic settings assume all individuals are exposed to TB [25], [32]. However, studies have shown individuals may be persistently exposed to Mtb but never develop LTBI [15], [19]. Characterization of controls in TB genetics studies has differed widely (examples in Table 1). Many studies have utilized population controls, similar to the approach taken in recent large genome-wide association studies (GWAS) [33], i.e., by using blood bank donors. The disadvantage of this design is possible misclassification bias [34]—the chance that some of these “controls” may never become affected for TB, which is problematic when the disease is common [35]. By contrast, other studies have utilized unaffected household members [26], [31], [36], [37] or have conducted thorough clinical evaluation with TST in those without disease [27], [30], [38]; in these situations, exposure in unaffected individuals is known, so these are true controls in the epidemiological sense. Note that only one of the *NRAMP1* associations was observed in studies where exposure has been quantified (Table 1).

### Epidemiological Study Design

The vast majority of genetic epidemiological studies, not just for TB but for other complex traits as well, tend to be case-control studies. Such studies are easier to conduct because they do not require cooperation of the entire family, and a greater number of cases can be recruited. One major advantage of family-based designs for the study of infectious diseases is the characterization of exposure in the “controls”, as discussed above. Individuals living in the same household have a high likelihood of exposure to an infectious TB case, thereby influencing the probability that they too will develop TB [39]–[41]. As described above, epidemiological characterization of exposure is important in order to construct a valid case-control study.

Another advantage of family-based studies is the ability to account for population substructure. Hidden population stratification may result in bias (false positive results) [42] or false negative results [43]. Studies of TB genetics have been conducted in many admixed populations, including African Americans [26], [27], [44]–[46], Mexicans [30], and South African “Coloureds” [11], [14], [32], [47], [48]. Some of these studies [11], [26], [45], [46] have employed family-based designs. Other studies have examined potential population substructure by analyzing genomic control markers: one study in South Africa utilized ∼25 markers [47], [48], and another study utilized >200 markers [49]. Marchini et al. [43] point out genomic control markers will not adequately correct for population substructure if too few markers are used, but it is difficult to enumerate a sufficient number of markers in populations of African descent. It is unclear if other studies were able to account for population substructure. It may be impossible for existing study cohorts to incorporate family-based designs or retrospectively evaluate population stratification, but this clearly may explain some of the heterogeneity among studies.

### Population Differences—More Than Just Geography

A typical explanation for differing results by population is population differentiation [22], [50], including genetic heterogeneity or inestimable polygenic effects. Another important genetic difference between populations is in linkage disequilibrium (LD).

Early studies of TB genetics were restricted to well-characterized markers within genes (studies of *SLC11A1/NRAMP1* in Table 1 are examples). Often these markers were exonic or restriction fragment length polymorphisms. The underlying assumption of the power and design of such studies is that the polymorphism being analyzed is the causal polymorphism.

**Figure 1:**
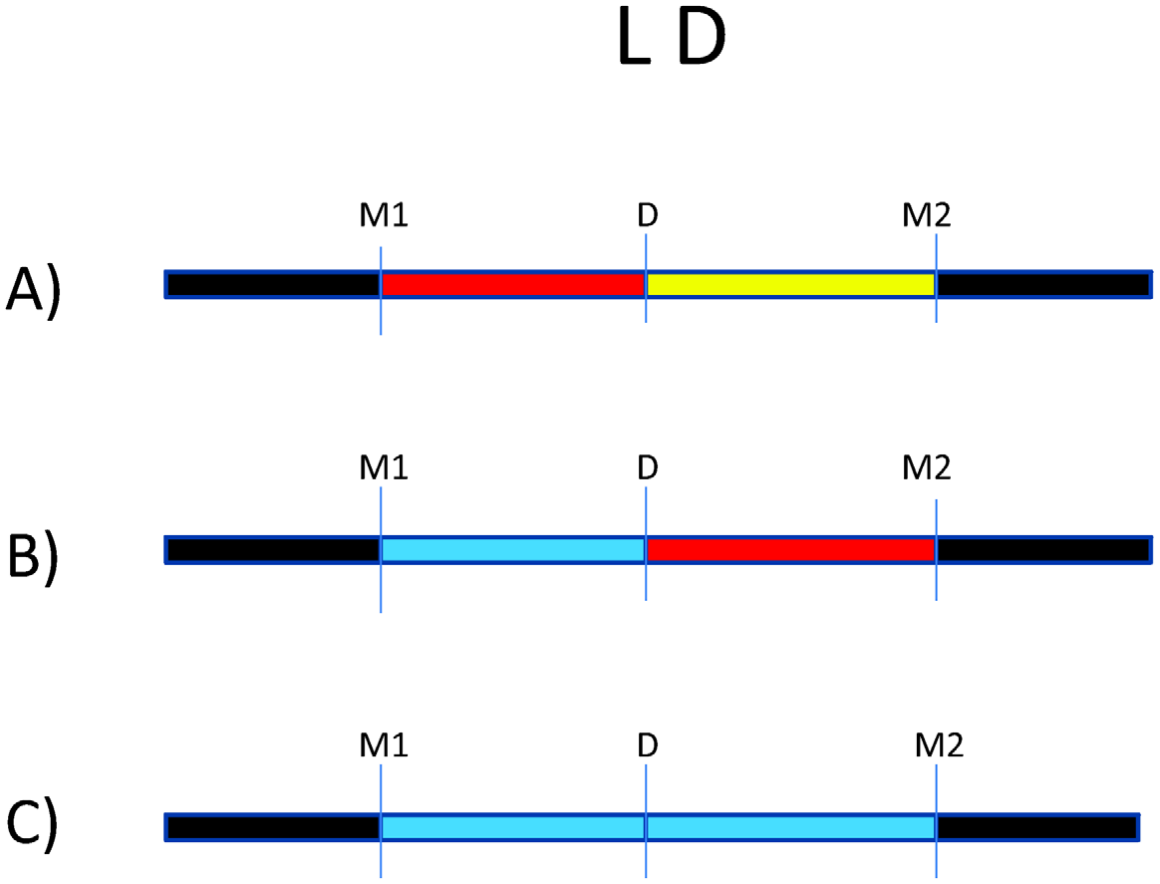
Impact of variation in linkage disequilibrium (LD) in detection of disease risk alleles. For all three scenarios, D is the underlying disease risk allele. (A) There is strong LD between D and marker #1 (M1), and weak LD between D and M2. In this situation, association will be detected with M1, depending on study power based on sample size, strength of genetic effect, and minor allele frequencies. (B) There is no LD between M1 and D but strong LD between M2 and D. Here, association will be detected only with M2 (again, depending on power). (C) There is weak LD throughout the region. Association will likely not be detected.

There are millions of single nucleotide polymorphisms (SNPs) throughout the genome [51], [52]. Because of the LD structure in the genome, certain SNPs can be used to “tag” haplotypes, such that one or a few SNPs capture information about LD structure [53]. Many trait-associated SNPs (>40%) are intergenic or intronic, suggesting an important role for non-coding SNPs in complex disease [54]. This serves as a reminder that disease risk alleles may actually be in LD with genotyped markers, which serve as “tags” for haplotypes on which the causal allele may reside. This is illustrated by Figure 1, where we consider an underlying disease allele that is not directly genotyped but surrounded by flanking markers. The ability to detect association with the region where the disease allele resides depends entirely on the strength of LD between the unobserved risk allele and flanking markers. As patterns of LD differ between study populations, the specific trait-associated SNPs will consequently differ.

**Figure 2:**
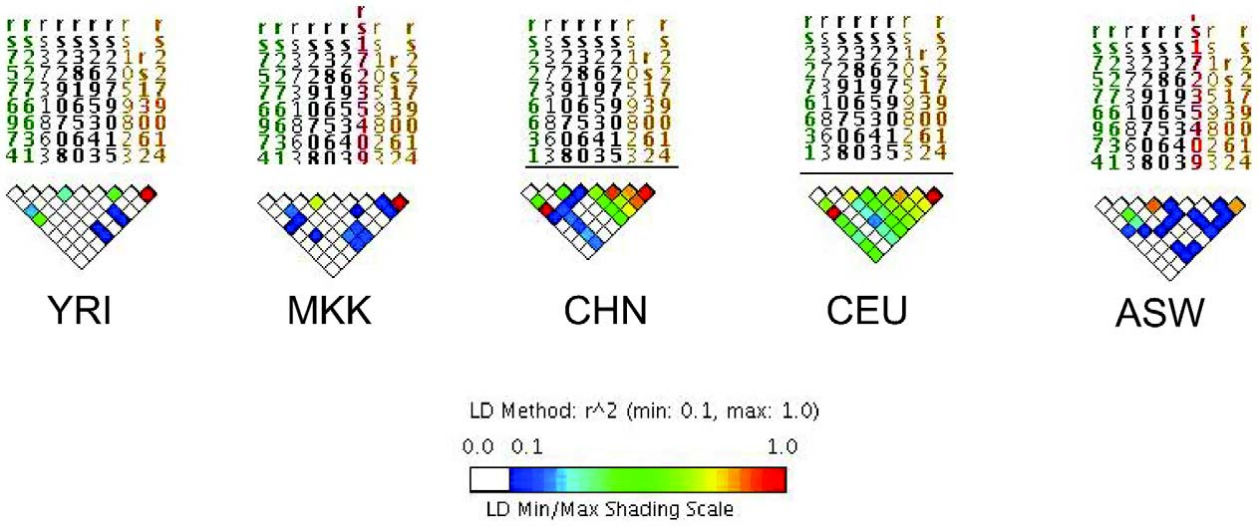
Linkage disequilibrium (LD) of the NRAMP1 gene for HapMap reference populations. Yoruba (YRI), Maasai (MKK), Han Chinese (CHN), Utah Caucasians (CEU), and African Americans (ASW) are shown. The strength of LD is illustrated using the color scale shown in the figure key.

The impact of LD differences between study populations is further illustrated in Figure 2. Here, LD patterns in *NRAMP1* were plotted using HapMap reference populations representative of those populations where NRAMP1 has been studied: Caucasians in Utah, United States (CEU), Yoruba in Nigeria (YRI), Maasai in Kenya (MKK), Han Chinese (CHN), and African Americans in the US Southwest (ASW). These LD plots were generated using default parameters in the Genome Variation Server (http://gvs.gs.washington.edu/GVS/), with no minor allele frequency cutoff. African populations (YRI and MKK) have very little LD because they are older populations, and their LD patterns differ. Newer populations (CEU and CHN) have much greater LD, and recently admixed populations (ASW) also exhibit LD between SNPs, but there are differences. Also note that the SNPs themselves (rs numbers) differ between populations, illustrating how different polymorphisms exist within the same genes across world populations. A perfect illustration of this phenomenon is provided by Velez et al. [26], who analyzed a number of SNPs within *NRAMP1*. Though they did not observe statistical association with the markers that were examined in early studies, they did observe association with intronic and exonic SNPs. If they had not conducted such extensive genotyping, they may have missed these associations.

Only a few other studies have accounted for global variations in LD by analyzing several SNPs within candidate genes of interest. Some studies have selected tag SNPs based on relevant HapMap reference populations [26], [28], [31], [46]. Other studies have sequenced genes of interest first to identify novel SNPs within the gene(s), then analyzed association with those SNPs [55]–[58]. Though other studies did not utilize LD in their selection of SNPs, they later estimated LD between markers in their dataset, and used this analysis to guide haplotype analysis [48], [49]. Since LD patterns differ by population, it should not be surprising that genetic association results differ, especially given the limited number of markers analyzed per gene. There are many implications of this variation. Differences in the strength of LD between the actual disease locus and genotyped markers will affect the power to detect association to markers (Figure 1). In populations with weaker LD such as African populations, denser SNP observed maps are necessary to detect association effects with untyped disease loci. Thus, variation in number of polymorphisms analyzed, differences in LD in the reference population, and existence of still-unknown risk alleles all complicate replication across studies.

Another controversial issue is the study of common versus rare genetic variants. The common disease–common variant (CDCV) hypothesis posits that genetic risk for common diseases will often be due to common risk alleles [59]. This is in contrast to the common disease rare variant (CDRV) hypothesis, which states that a significant proportion of common chronic diseases are influenced by the summation of effects of multiple low frequency variants in the same gene, where tagging SNPs will not be useful in identifying a single haplotype because no single haplotype exists [60]. Most candidate gene studies assume the CDCV hypothesis. Recent sequencing studies [61], [62] have detected rare SNPs in the TLR family of genes; these could be important, but massive studies will be needed in order to detect disease associations at a statistically significant threshold. In addition, copy number variants (CNVs) have recently attracted attention in their association with complex traits, such as HIV acquisition and progression and autoimmune diseases [63]. These are also considered rare variants, so we are again faced with all of the challenges of testing the CDRV hypothesis.

The above discussion focuses on population genetics of humans. Another related issue is variation in Mtb strains. Researchers have categorized Mtb into six main bacterial strain lineages that are associated with particular geographical regions [64], as well as differences in clinical presentation [65] and rate of progression to active TB disease [66]. So, not only do different diagnostic criteria, as discussed above, potentially reflect differences in disease severity, but specific Mtb strains may also influence disease severity. A recent study suggests a host genotype x Mtb genotype interaction, whereby the *TLR2* genotype is associated with TB caused by the Beijing strain [67]. Very few studies have the capacity to examine this potential host by Mtb interaction, but it could easily be a potential explanation for differences between studies.

### Complex Genetic Effects

Complex traits such as TB are likely influenced by several factors, including gene–gene interaction and gene–environment interaction. Few studies have investigated gene-gene interactions in the context of human TB. Many gene products (e.g., Toll-like receptors [TLRs]) are known to interact biologically [68], and interaction effects have been demonstrated in mouse models of TB [69]. A recent study identified interactions between the *NOS2A* gene and *IFNGR1* and *TLR4* [45]. Interestingly, both *IFNGR1* and *TLR4* showed no evidence of significant main effects in this analysis. Another study by the same research group found interaction between *NRAMP1* and *TLR2*, but *TLR2* did not itself have a significant main effect [26]. This suggests many important genes may influence TB in combination with other genes, but this could be overlooked because their individual effects did not meet criteria for statistical significance. Motsinger-Reif et al. used multifactor dimensionality reduction to identify a potential gene–gene interaction between *TLR4* and the TNF-α gene (*TNF*) [70]. In addition, it is well known that HIV influences the pathogenesis of TB, but most genetic epidemiological studies have been restricted to HIV seronegative individuals. Our work [31] showed an interaction between HIV and the TNF receptor 1 gene. Because many studies have excluded HIV-positive individuals, this hypothesis remains relatively unexplored. Similar to the TNF-α pathway, the type I and II interferon pathways have been associated with both TB and HIV pathogenesis [71], and so should also be considered for future studies of gene–HIV interactions. The challenge of examining interaction effects is the requirement of even larger sample sizes, as discussed by Velez et al. [45].

## Conclusions, Recommendations, and Future Directions

As reviewed recently by Möller et al. [22], the body of work showing statistical associations between candidate genes and TB continues to grow. This does not include potential unpublished studies that failed to find significant associations and are not readily available due to publication bias [22]. Even in the published body of literature, however, there is a great deal of inconsistency between marker-trait associations, so we are far from reaching a consensus regarding genes involved in TB risk.

This review focused on methodological reasons for inconsistency across studies. One important factor is the diagnostic criteria for TB disease, which have differed dramatically across studies. Resources available for TB diagnosis differ by country, which is confounded when there has been conflict [72]. Differences in diagnostic criteria across studies can reflect differences in TB severity and may lead to misclassification of cases as controls; this would have a significant impact on the type I and type II error of studies. It is impossible to standardize the diagnostic definitions used across all study sites, but researchers should be mindful of such differences when interpreting their findings. We strongly recommend that researchers characterize the level of exposure to Mtb in individuals without disease, which should include TST/IGRA and careful epidemiological characterization. New studies could utilize the household contact design, which facilitates the characterization of all stages of Mtb exposure, infection, and disease [41]. When the household contact study design is not feasible, spousal controls are also ideal because of persistent and prolonged exposure.

Recall that TB follows two stages of pathogenesis, and LTBI precedes TB disease. Recent studies suggest that LTBI may have unique genetic influences [15], [28], [29]. Persons with LTBI constitute a major impediment to TB control efforts [73]. Since many ongoing vaccine development efforts will focus either preventing LTBI or progression to TB, it is important to understand host factors that influence containment of Mtb infection. However, the study of the genetics of LTBI is also not trivial. Indication of T cell memory response via positive TST and/or IGRA does not necessarily imply the presence of viable Mtb bacilli. In the US as well as other public health systems, individuals with positive TST are treated as though there are viable organisms present, adding further confusion to this phenotype. According to Parrish et al., there is a 2%–23% lifetime probability of developing TB after acquisition of Mtb infection (LTBI) [73]. This illustrates the heterogeneity in this clinical group, since the risk of progression to active TB may depend on a variety of known and unknown risk factors. Furthermore, prophylaxis of LTBI with isoniazid (INH) is the standard of care in many research settings, so that many individuals with “LTBI” based on positive TST/IGRA, genetically predisposed to develop TB, may not. One way to investigate the role of host genetics in LTBI would be to compare TST (or IGRA) positive individuals that develop incident TB to those that do not. Ideally, such a study would not include individuals on INH prophylaxis, though that is unethical in many settings. For these reasons, some may argue that it is more relevant to study TB genetics, and not LTBI, from a public health standpoint.

Thus, it is essential to take a multidisciplinary approach [74] to develop an all-encompassing picture of the natural history of Mtb infection and disease. Few studies have examined the genetics of TB immunology [15], [31], [75]–[77]. Gene expression studies using microarrays may also shed light on host responses to Mtb [78]. Proteomic studies will further elucidate host factors involved in pathogenesis. These various approaches should be analyzed together to hopefully identify more meaningful clinical groups. For example, genomic, proteomic, and immunologic data, collectively, may better capture the heterogeneity in latently infected individuals.

Additional complicating factors in comparing geographically diverse studies are potential population substructure and LD differences among populations. We recommend that future studies analyze enough SNPs to capture LD in their study population. Analyses of a few markers within a gene no longer advance the field, particularly in light of LD differences between populations. Even with advances in genotyping, many studies of “old” markers continue to be published. The choice of a reference population for tag SNP selection is not trivial [62]; thus, dense SNP mapping may be necessary, particularly in studies of African populations. If it is impossible to rigorously examine genes in this way, publishing the LD patterns in the study data [28], [45], [48], [49] is a good start. Furthermore, studies in admixed populations should attempt to examine population substructure to minimize this source of bias. Populations also differ in the Mtb strain lineage that caused TB; future studies examining host gene by Mtb gene interaction are warranted. Finally, as in all genetic epidemiological studies of complex traits, genes may act in complex ways. Genes may interact with other genes and/or epidemiological factors; these potential relationships should not be overlooked. Furthermore, too many researchers (authors and journal reviewers alike) focus too much on *p*-values. All *p*-values must be reported, even if greater than 0.05. Markers with *p*-values greater than 0.05 may still be important in their interaction with other markers or environmental factors. Researchers should collect sufficient data to explore these meaningful biological effects.

There are GWAS of TB forthcoming. Given the issues discussed in this review, we must interpret the findings of those GWAS cautiously. Will these studies be underpowered due to the heterogeneity among TB cases and controls? A recent summary analysis of published GWAS found the reported SNP–trait associations attaining significance (*p*<10^−5^) had a median odds ratio of 1.33, with an interquartile range of 1.20–1.61 [54]; thus, the effect sizes of SNPs identified through GWAS are relatively small. Furthermore, the proportion of heritability explained by these variants ranges between 1% and 50% [79]. TB GWAS may provide new clues into the host biology of TB pathogenesis, but the overall clinical relevance of these SNPs will be limited. In addition, GWAS of other complex traits have often merged data across ongoing research studies. Because of the dramatic heterogeneity among studies described in this review, meta-analyses of TB genetic association studies should be conducted with care.

In sum, we have barely scratched the surface in understanding the genetic determinants of TB pathogenesis. Because of the significant public health impact of TB, additional studies are necessary, and should be multidisciplinary in nature. Future studies should carefully consider phenotype definition and genetic epidemiological principles when designing, analyzing, and interpreting findings. Ideally, culture confirmation for pulmonary TB should be conducted where feasible, thorough epidemiological data should be collected in individuals without TB to better understand LTBI and risk of progression to TB, and population genetic factors should be carefully characterized and considered in the analysis.

### Accession Numbers for Genes Mentioned in This Paper (GeneIDs from EntrezGene)

*TLR2* (7097); *SLC11A1*, aka *NRAMP1* (6556); *IFNGR1* (3459); *TLR4* (7099); *TNF* (7124); *TNFSF1A*, aka TNF receptor 1 (7132); *NOS2A* (4843).
